# Hemorrhage of brain metastasis is a poor prognostic factor in hepatocellular carcinoma patients

**DOI:** 10.18632/oncotarget.21449

**Published:** 2017-10-03

**Authors:** Yan Lin, Shi-Ting Huang, Yan-Ming Jiang, Xin-Bin Pan

**Affiliations:** ^1^ Department of Radiation Oncology, Cancer Hospital of Guangxi Medical University, Nanning, Guangxi, P.R. China; ^2^ Department of Gastroenterology, The Third People's Hospital of Guangxi Zhuang Autonomous Region, Nanning, Guangxi, P.R. China

**Keywords:** hepatocellular carcinoma, brain metastasis, hemorrhage

## Abstract

It is unclear whether hemorrhage of brain metastasis is a poor prognostic factor in patients with hepatocellular carcinoma. We conducted a retrospective cohort study to compare overall survival between hemorrhage and no-hemorrhage groups of hepatocellular carcinoma patients with brain metastasis. Hepatocellular carcinoma patients with brain metastasis treated between June 2000 and June 2016 at the Cancer Hospital of Guangxi Medical University were retrospectively reviewed. Clinical characteristics and overall survival were compared between patients with (*n* = 11) and without (*n* = 25) hemorrhage of brain metastasis. Univariate and multivariate survival analyses showed hemorrhage to be a poor prognostic factor (hazard ratio = 5.812, 95% confidence interval: 1.399-24.142, *p* = 0.015). Patients with hemorrhage had a shorter median survival than those without hemorrhage (4 weeks vs 8 weeks, *p* = 0.001). These results suggest hemorrhage of brain metastasis is a poor prognostic factor in patients with hepatocellular carcinoma patients.

## INTRODUCTION

Hepatocellular carcinoma (HCC) is one of the most commonly occurring cancers in Southeast Asia [1]. In China, it is also one of the top three causes of cancer death in areas where hepatitis B infections are prevalent. Moreover, the incidence of HCC is rising in Western countries [2]. The lungs, bone, and adrenal glands are common metastasis sites in HCC, whereas brain metastasis (BrM) is rare [2], with an incidence of 0.2% to 2.2% [3–9]. Because the prognosis of HCC patients with BrM is extremely poor [3, 5–7, 10], prognostic factors and treatment modalities are not well defined.

BrM from HCC is fast growing, highly vascularized, and commonly associated with hemorrhage [11], though several studies suggest hemorrhage is not a prognostic factor in HCC and does not affect survival duration [5, 6, 10, 12]. On the other hand, Han et al [7] reported that hemorrhage of BrM was associated with poor overall survival in HCC.

Recent therapeutic advances for HCC have contributed to improved survival rates [2]. As a result, the incidence of BrM is expected to increase as HCC patients survive longer [4]. We therefore conducted a retrospective cohort study to assess the prognosis of HCC patients with hemorrhage from BrM. We anticipate the results of this study may help clinicians make better treatment decisions for HCC patients.

## RESULTS

A total of 39 HCC patients were diagnosed with BrM. Three of those were excluded because of incomplete data, so this study ultimately included 36 patents. All of these had patients died by the final follow-up. Table 1 summarizes patients’ characteristics.

**Table 1:** Characteristics of 36 HCC patients with BrM

	Total (*n* = 36)	No hemorrhage (*n* = 25)	Hemorrhage (*n* = 11)	*P*
**HCC characteristics**				
Age at BrM diagnosis (years, Mean±SD)	47.25±11.23	46.08±11.18	49.91±11.40	0.353
Interval from HCC to BrM (months, M(Q1,Q3)	5.5 (0, 19)	5.0 (0, 13)	9.0 (0, 20)	0.972
Sex				0.216
male	33 (91.67%)	24 (96.00%)	9 (81.82%)	
female	3 (8.33%)	1 (4.00%)	2 (18.18%)	
KPS				0.624
<100	1 (2.78%)	1 (4.00%)	0 (0.00%)	
<90	5 (13.89%)	4 (16.00%)	1 (9.09%)	
<80	27 (75.00%)	19 (76.00%)	8 (72.73%)	
<70	3 (8.33%)	1 (4.00%)	2 (18.18%)	
Hepatitis B				0.224
positive	26 (72.22%)	20 (80.00%)	6 (54.55%)	
negative	10 (27.78%)	5 (20.00%)	5 (45.45%)	
AFP				0.446
>400	24 (66.67%)	18 (72.00%)	6 (54.55%)	
≤400	12 (33.33%)	7 (28.00%)	5 (45.45%)	
Child-Pugh's classification				1.000
A	18 (50.00%)	12 (48.00%)	6 (54.55%)	
B	16 (44.44%)	11 (44.00%)	5 (45.45%)	
C	2 (5.56%)	2 (8.00%)	0 (0.00%)	
RPA class				0.463
I	1 (2.78%)	1 (4.00%)	0 (0.00%)	
II	32 (88.89%)	23 (92.00%)	9 (81.82%)	
III	3 (8.33%)	1 (4.00%)	2 (18.18%)	
Primary tumor				0.678
uncontrolled	28 (77.78%)	20 (80.00%)	8 (72.73%)	
controlled	8 (22.22%)	5 (20.00%)	3 (27.27%)	
Extracranial metastasis				0.352
none	15 (41.67%)	9 (36.00%)	6 (54.55%)	
single	17 (47.22%)	12 (48.00%)	5 (45.45%)	
multiple	4 (11.11%)	4 (16.00%)	0 (0.00%)	
HCC treatment				0.781
resection	12 (33.33%)	8 (32.00%)	4 (36.36%)	
TACE	13 (36.11%)	10 (40.00%)	3 (27.28%)	
RFA	3 (8.33%)	1 (4.00%)	2 (18.18%)	
radiotherapy	1 (2.78%)	1 (4.00%)	0 (0.00%)	
chemotherapy	1 (2.78%)	1 (4.00%)	0 (0.00%)	
palliative	6 (16.67%)	4 (16.00%)	2 (18.18%)	
**BrM characteristics**				
Symptoms				0.394
headache	13 (36.11%)	10 (40.00%)	3 (27.27%)	
mental status changes	2 (5.56%)	1 (4.00%)	1 (9.09%)	
nausea	2 (5.56%)	2 (8.00%)	0 (0.00%)	
aphasia	1 (2.78%)	1 (4.00%)	0 (0.00%)	
visual disturbance	1 (2.78%)	0 (0.00%)	1 (9.09%)	
cerebellar dysfunction	1 (2.78%)	0 (0.00%)	1 (9.09%)	
none	16 (44.43%)	11 (44.00%)	5 (45.46%)	
Signs				0.597
motor disturbance	11 (30.56%)	7 (28.00%)	4(36.36%)	
none	25 (69.44%)	18 (72.00%)	7 (63.64%)	
Location				0.280
parietal	14 (38.88%)	11 (44.00%)	3 (27.28%)	
occipital	6 (16.67%)	2 (8.00%)	4 (36.36%)	
temporal	2 (5.56%)	2 (8.00%)	0 (0.00%)	
cerebellar	1 (2.78%)	1 (4.00%)	0 (0.00%)	
frontal	4 (11.11%)	2 (8.00%)	2 (18.18%)	
multiple locations	9 (25.00%)	7 (28.00%)	2 (18.18%)	
Number				0.690
single	27 (75.00%)	18 (72.00%)	9 (81.82%)	
multiple	9 (25.00%)	7 (28.00%)	2 (18.18%)	
BrM treatment				0.395
resection + WBRT	3 (8.33%)	3 (12.00%)	0 (0.00%)	
resection	3 (8.33%)	1 (4.00%)	2 (18.18%)	
SRS	3 (8.33%)	3 (12.00%)	0 (0.00%)	
WBRT	2 (5.56%)	2 (8.00%)	0 (0.00%)	
chemotherapy	1 (2.78%)	1 (4.00%)	0 (0.00%)	
palliative (Steroid alone)	24 (66.67%)	15 (60.00%)	9 (81.82%)	

HCC: hepatocellular carcinoma, BrM: brain metastasis, SD: standard deviation, KPS: performance status, AFP: alpha fetoprotein, RPA: recursive partitioning analysis, TACE: transcatheter arterial chemoembolization, RFA: radiofrequency ablation, WBRT: whole brain radiotherapy, SRS: stereotaxic radiosurgery.

**Figure 1:**
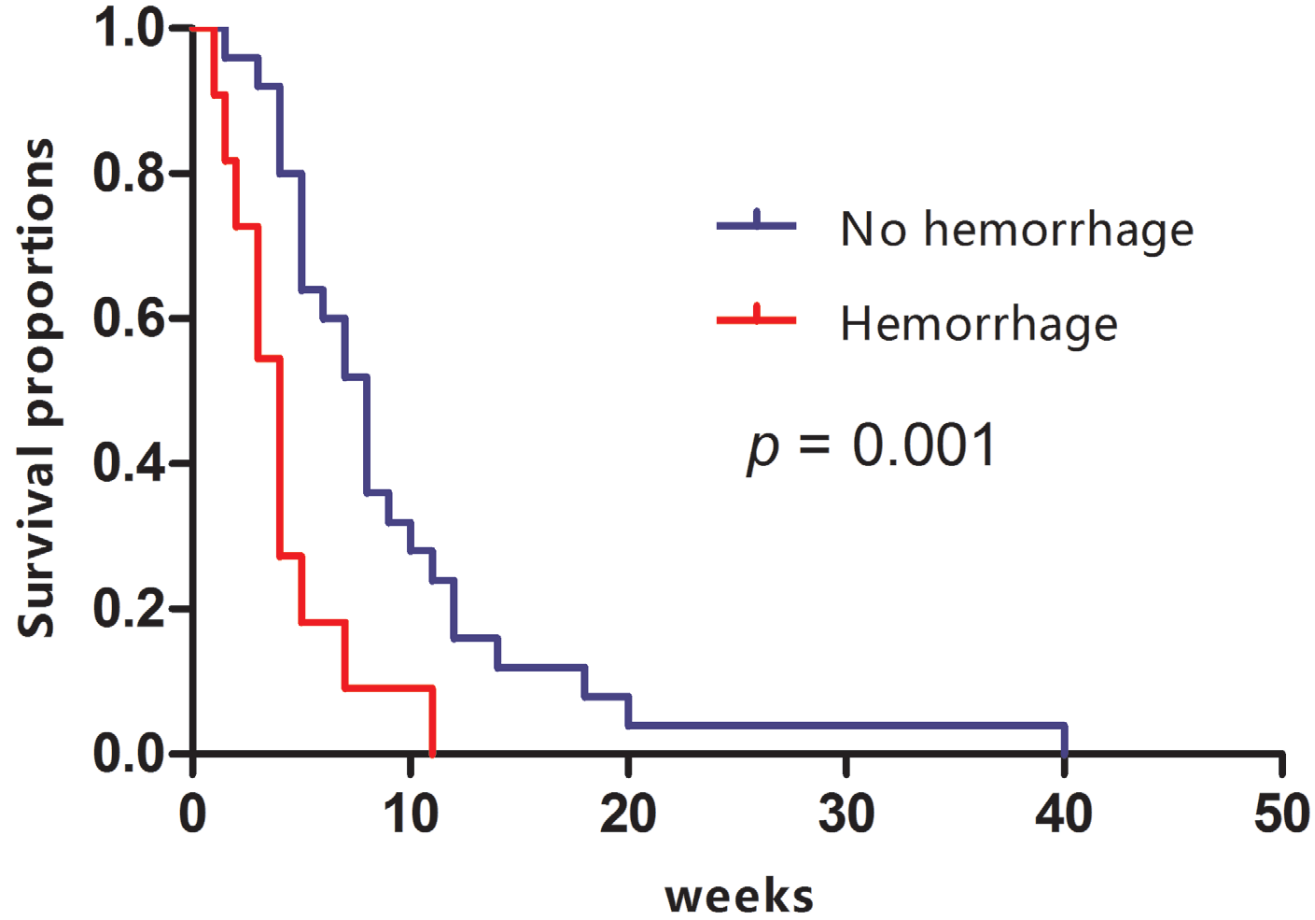
Kaplan-Meier curves comparing survival between the hemorrhage and no-hemorrhage groups of HCC patients with BrM

Figure 1 shows the comparison of overall survival between the hemorrhage and no-hemorrhage groups. In a univariate analysis, the variables correlated with median survival after diagnosis of BrM were RPA, HCC treatment modality, hemorrhage, and BrM treatment modality (Table 2). The results showed that there was a significant difference in median survival between the hemorrhage and no-hemorrhage groups (4 weeks vs 8 weeks, *p* = 0.001). To correct for possible confounding factors, we used multivariate logistic regression to assess the effect of hemorrhage. We found that hemorrhage of BrM was indeed a poor prognostic factor affecting median survival (hazard ratio [HR] = 5.812, 95% confidence interval [CI]: 1.399-24.142, *p* = 0.015).

**Table 2:** Univariate and multivariate analyses for survival predictors in HCC patients with BM

Variables	No	Median survival (weeks)	Univariate (P)	Multivariate		
				HR	95% CI	*P*
**HCC characteristics**						
Age when BrM developed						
≥47 years	18	7	0.414	1.042	0.970-1.121	0.261
<47 years	18	5				
Interval from HCC to BrM						
>5 months	18	5	0.778	1.006	0.983-1.030	0.620
≤5 months	18	5				
Sex						
male	33	6	0.266	0.727	0.076-6.991	0.782
female	3	4				
KPS						
≥80	6	8	0.302	0.883	0.127-6.129	0.900
<80	30	5				
Hepatitis B						
positive	26	5	0.836	1.221	0.267-5.587	0.797
negative	10	5				
AFP						
>400	24	5	0.953	1.113	0.221-5.612	0.896
≤400	12	5				
Child-Pugh's classification						
A	18	7	0.480	1.128	0.503-2.528	0.770
B	16	5				
C	2	4				
RPA class						
III	3	1	0.000	38.422	2.347-629.090	0.011
II	32	6				
I	1	14				
Primary tumor						
uncontrolled	28	5	0.843	0.686	0.109-4.300	0.687
controlled	8	5				
Extracranial metastasis						
none	15	7	0.561	2.012	0.793-5.107	0.141
single	17	6				
multiple	4	4				
HCC treatment						
palliative	6	3	0.045	1.616	0.413-6.317	0.491
HCC treated	30	6		1		
**BrM characteristics**						
Symptoms						
yes	20	5	0.812	0.503	0.126-2.013	0.332
no	16	6				
Signs						
yes	11	4	0.096	2.923	0.531-16.095	0.218
no	25	7				
Number						
single	27	7	0.689	0.662	0.248-1.761	0.408
multiple	9	5				
Hemorrhage						
yes	11	4	0.001	5.812	1.399-24.142	0.015
no	25	8		1		
BrM treatment						
palliative (Steroid alone)	24	4	0.000	28.601	6.329-129.255	0.000
BrM treated	12	11		1		

HCC: hepatocellular carcinoma, BrM: brain metastasis, KPS: performance status, AFP: alpha fetoprotein, RPA: recursive partitioning analysis, HR: hazard ratio, CI: confidence interval.

HCC treated: HCC treated with resection, transcatheter arterial chemoembolization, radiofrequency ablation, radiotherapy, or chemotherapy.BM treated: BM treated with resection, whole brain radiotherapy, stereotaxic radiosurgery, or chemotherapy.

BM treated: BM treated with resection, whole brain radiotherapy, stereotaxic radiosurgery, or chemotherapy.

## DISCUSSION

Our study suggests that HCC patients with BrM hemorrhage have a poorer prognosis than those without hemorrhage. This finding suggests clinicians should pay greater attention to BrM hemorrhage when making treatment decisions.

Previous studies reported that BrM from HCC is frequently associated with hemorrhage [5–7, 10, 12], with incidences of 41.94% to 74.74%. In the present study, the hemorrhage rate among HCC patients with BrM was 30.56%. At our hospital, brain imaging is not performed only in cases with neurologic symptoms/signs, but also part of the routine evaluation of HCC patients. Consequently, 12 patients in this study were diagnosed with BrM at the time of their HCC diagnosis, which may account for the lower rate of BrM hemorrhage in our study.

Whether BrM hemorrhage significantly affects survival in HCC patients is controversial. Univariate and multivariate analyses carried out in several studies have suggested that BrM hemorrhage is not a prognostic factor associated with difference in survival [5, 6, 10]. For example, Hsieh et al [12] reported that the occurrence of BrM hemorrhage did not influence overall survival of HCC patients as compared to patients who did not experience BrM hemorrhage. By contrast, Han et al [7] reported that BrM hemorrhage was predictive of poorer prognosis, as patients without hemorrhage survived longer than who experienced BrM hemorrhage (13.7 weeks vs 8.1 weeks, *p* = 0.044 in univariate analysis). Both our univariate and multivariate analyses also indicate BrM hemorrhage is a poor prognostic factor and that HCC patients with BrM hemorrhage have a significantly shorter median survival than those without hemorrhage. In our study, 81.82% patients with BrM hemorrhage received palliative care. This may explain the poorer survival compared to earlier studies [6, 7], as patients who received palliative care had a poorer prognosis than those who received therapeutic treatment. This would confound the result in the context of a treatment effect versus patient selection effect.

In this study, palliative care was associated with poorer survival than BrM treatment, including resection, whole brain radiotherapy, stereotaxic radiosurgery, or chemotherapy (4 weeks vs 11 weeks, *p* = 0.001). However, the best treatment modalities for BrM from HCC are not clear due to its rarity. The treatment may be similar to the general guidelines for metastatic brain tumors. For a single large lesion (<3 cm), surgical resection or stereotaxic radiosurgery should be considered with/without whole brain radiotherapy. Surgery was also a good treatment option for hemorrhagic BrM, though increased intracranial pressure and severe neurologic deficits may have existed [13]. In our study, two patients with BrM hemorrhage received resection, and they showed considerably prolonged survival (7 and 11 weeks). However, hemorrhage can lead to severe neurological deficits and poor functional status. Moreover, poor liver function may lead to underlying coagulopathy. Surgery is restricted in most HCC patients with BrM hemorrhage, making radiotherapy the preferred treatment modality. Stereotactic body radiation therapy and stereotaxic radiosurgery are effective for controlling BrM, especially when there is intratumoral hemorrhage [5, 6]. In sum, decisions about treatment of BrM from HCC should be made cautiously, especially in patients with poor RPA class and/or KPS.

This study had the following limitations. (1) Only 36 patients were enrolled in our study, and the sample size of the hemorrhage group was small. (2) In retrospective cohort studies, exclusion of potential biases is difficult. Patients included in our study varied with regard to KPS, extracranial metastasis, HCC treatment, and BrM treatment. Consequently, confounding factors could be inherent in this study. Further large-scale studies are necessary to verify the results.

In conclusion, this study suggests that BrM hemorrhage is a poor prognostic factor for HCC patients.

## MATERIALS AND METHODS

This study was approved by the Ethics Committee of the Cancer Hospital of Guangxi Medical University. HCC patients treated between June 2000 and June 2016 at the Cancer Hospital of Guangxi Medical University were retrospectively reviewed. HCC was diagnosed based on pathology or radiological criteria [2]. BrM was diagnosed based on computerized tomography (CT) and/or magnetic resonance imaging (MRI), with or without pathology.

Clinical data at the time BrM was diagnosed, including age, sex, time interval from HCC diagnosis to BrM, Karnofsky performance status (KPS), Child-Pugh classification, recursive partitioning analysis (RPA) class, level of alpha fetoprotein (AFP), and extracranial metastasis, were collected. Also evaluated were data on BrM, including presenting symptoms/signs, location, number, hemorrhage, treatment modality, and survival time. BrM hemorrhage was diagnosed based on the pathology at surgery and/or CT/MRI. Patients were divided into hemorrhage and no-hemorrhage groups. The endpoint of this study was overall survival. The follow-up period was terminated by death or the beginning of this study (March 2017).

Categorical variables were compared using the Chi-square test or Fisher's exact t-test. Continuous variables were expressed as the mean ± standard deviation and compared using Student's t-test. Prognostic factors were analyzed using log-rank test for univariate analysis; Cox regression analysis was used for multivariate analysis. Overall survival was calculated from the diagnosis of BrM of death or last day of follow using the Kaplan-Meier method and compared using the log-rank test.

Statistical analysis was performed using SPSS for Windows version 16.0 (SPSS Inc., Chicago, IL). All tests were two-sided, and values of P <0.05 were considered statistically significant.

## Abbreviations

HCC: hepatocellular carcinoma
BrM: brain metastasis
CT: computerized tomography
MRI: magnetic resonance imaging
KPS: Karnofsky performance status
RPA: recursive partitioning analysis
AFP: alpha fetoprotein

## References

[1] Jemal A, Bray F, Center MM, Ferlay J, Ward E, Forman D (2011). Global cancer statistics. CA Cancer J Clin.

[2] Rahbari NN, Mehrabi A, Mollberg NM, Muller SA, Koch M, Buchler MW, Weitz J (2011). Hepatocellular carcinoma: current management and perspectives for the future. Ann Surg.

[3] Choi HJ, Cho BC, Sohn JH, Shin SJ, Kim SH, Kim JH, Yoo NC (2009). Brain metastases from hepatocellular carcinoma: prognostic factors and outcome: brain metastasis from HCC. J Neurooncol.

[4] Seinfeld J, Wagner AS, Kleinschmidt-DeMasters BK (2006). Brain metastases from hepatocellular carcinoma in US patients. J Neurooncol.

[5] Zhang RL, Zhang H, Zhang L, Xiao L, Sun YN, Yang Y, Bao YX (2016). [Brain metastases from hepatocellular carcinoma: clinical features and prognostic factors in 31 cases]. [Article in Chinese]. Zhonghua Zhong Liu Za Zhi.

[6] Jiang XB, Ke C, Zhang GH, Zhang XH, Sai K, Chen ZP, Mou YG (2012). Brain metastases from hepatocellular carcinoma: clinical features and prognostic factors. BMC Cancer.

[7] Han MS, Moon KS, Lee KH, Cho SB, Lim SH, Jang WY, Jung TY, Kim IY, Jung S (2013). Brain metastasis from hepatocellular carcinoma: the role of surgery as a prognostic factor. BMC Cancer.

[8] Murakami K, Nawano S, Moriyama N, Sekiguchi R, Satake M, Fujimoto H, Ichikawa T (1996). Intracranial metastases of hepatocellular carcinoma. CT and MRI. Neuroradiology.

[9] Kim M, Na DL, Park SH, Jeon BS, Roh JK (1998). Nervous system involvement by metastatic hepatocellular carcinoma. J Neurooncol.

[10] Kim KS, Kim K, Chie EK, Kim YJ, Yoon JH, Lee HS, Ha SW (2014). Prognostic stratification of brain metastases from hepatocellular carcinoma. J Neurooncol.

[11] Cheng SY, Nagane M, Huang HS, Cavenee WK (1997). Intracerebral tumor-associated hemorrhage caused by overexpression of the vascular endothelial growth factor isoforms VEGF121 and VEGF165 but not VEGF189. Proc Natl Acad Sci USA.

[12] Hsieh MJ, Lu CH, Tsai NW, Lui CC, Chuang YC, Huang CR, Chen SF, Chang CC, Chang HW, Chang WN (2009). Prediction, clinical characteristics and prognosis of intracerebral hemorrhage in hepatocellular carcinoma patients with intracerebral metastasis. J Clin Neurosci.

[13] Yoo H, Jung E, Gwak HS, Shin SH, Lee SH (2011). Surgical outcomes of hemorrhagic metastatic brain tumors. Cancer Res Treat.

